# Tobacco Smoking Status and Perception of Health among a Sample of Jordanian Students

**DOI:** 10.3390/ijerph110707022

**Published:** 2014-07-11

**Authors:** Sukaina Alzyoud, Khalid A. Kheirallah, Linda S. Weglicki, Kenneth D. Ward, Abdallah Al-Khawaldeh, Ali Shotar

**Affiliations:** 1 Department of Community and Mental Health, Faculty of Nursing, Hashemite University, P.O. Box 150459, Zarqa 13115, Jordan; 2 Department of Public Health, Faculty of Medicine, Jordan University of Science and Technology, P.O. Box 3030, Irbid 22110, Jordan; E-Mail: kkheiral@gmail.com; 3 College of Nursing, Wayne State University, Detroit, MI 48202, USA; E-Mail: weglickils@mail.nih.gov; 4 Division of Social and Behavioral Sciences, School of Public Health, University of Memphis, Memphis, TN 38152, USA; E-Mail: kdward@memphis.edu; 5 Department of Legal Medicine, Toxicology and Forensic science, School of Medicine, Jordan University of Science and Technology (JUST), P.O. Box 3030, Irbid 22110, Jordan; E-Mail: ashotar@just.edu.jo

**Keywords:** Jordan, tobacco, adolescent, smoking, perception, health

## Abstract

Limited data are available from Jordan examining patterns of tobacco use among adolescents, or how use is related to health perceptions. This study aims to estimate the prevalence of tobacco use and to assess the relationship between use and health-related perceptions. A cross-sectional survey was conducted among a sample of 11–18 year old school students from a major governorate in Jordan. Using a multistage random sampling 1050 students were selected. Students were categorized as non-smokers, cigarette-only smokers, waterpipe-only smokers, or dual smokers. Rates of waterpipe-only and cigarette-only smoking were 7% and 3%, respectively, and were similar for boys and girls. In contrast, the rate of dual use was much higher than for single product use and was double in girls compared to boys (34% *vs.* 17%). Dual-smokers were significantly more likely to think that it is safe to smoke as long as the person intends to quit within two years compared to non-smokers, and had lower self-rated health status than other groups. This is the first study among Arab adolescents to document high rates of dual tobacco use, especially pronounced among girls. The study findings have significant implications for designing tobacco smoking prevention programs for school health settings.

## 1. Introduction

Although tobacco use has declined in many high income countries such as the United States and United Kingdom, it is increasing in many low and middle income countries [1,2]. The World Health Organization (WHO) estimates there are more than 1.1 billion smokers worldwide, with more than 80% of them living in low and middle-income countries [3]. Especially problematic is that tobacco use, including forms of use other than cigarettes, is on the rise among adolescents in many countries, and is likely to jeopardize progress in reducing chronic diseases and tobacco-related mortality [2,4]. 

The Eastern Mediterranean region (EMR), consisting mainly of Arab countries, is one region that continues to experience an escalating tobacco epidemic. For example, between 1990 and 1997, cigarette consumption increased 24% in this region, while most other regions did not witness such a trend [5]. The EMR, moreover, is threatened by a reemergence of waterpipe tobacco smoking (a.k.a., hookah, shisha, narghile) [6].

Waterpipe is a centuries-old tobacco use method with origins in the Middle East and Indian subcontinent that had declined through much of the 20th century, but experienced a resurgence during the 1990s [7]. Tobacco use, particularly waterpipe, has also increased in recent years especially among youth [4,5,8,9,10,11]. Adolescents and young adults have been especially likely to take up the waterpipe, thought to be due to the growing availability of flavored and easy to use tobaccos known as *maa’sel* [8], a rapidly expanding “café culture” where waterpipe use is a popular and inexpensive way to socialize, and misperceptions that waterpipe is less harmful and addictive than cigarettes [9]. A recent systematic review of waterpipe prevalence studies across multiple countries and age groups reported that current use was highest among school students, especially in the United States, the Arabic Gulf region, Estonia, and Lebanon [10]. While cigarette smoking by women and girls in Middle Eastern countries has historically been very low [4,12], there is concern that waterpipe use is growing among adolescent and young adult females, for whom it is less stigmatized than cigarettes [6].

In Jordan, data on the prevalence and determinants of tobacco use among adolescents is scarce. There are only two reported studies; the first is the Jordan Global Youth Tobacco Survey (GYTS) which estimated the prevalence of ever smoking cigarettes among Jordanian youth, 13 to 15 years of age, as 39% in 2009 [4]. Waterpipe tobacco use was measured only in the 2009 Jordan GYTS, and overall current use prevalence was estimated as 21% (Boys = 27.1%, Girls = 15.6%; [4]). 

The second study was conducted by Mzayek and colleagues [13] who followed a sample of adolescents in Irbid, Jordan (*n* = 1702) from the 7th to the 9th grade (median age 13 years at the start of the study) to examine uptake of cigarette and waterpipe smoking. Among both boys and girls, the prevalence of ever smoking and current smoking of cigarettes and waterpipe use increased over two years, but was higher among boys at both time points. Current cigarette smoking increased from 8.6% to 23.1% among boys, and 1.8% to 6.4% among girls. Current waterpipe use was more prevalent than cigarette use during both 7th and 9th grades, and increased by 28% among boys (19.1% to 24.5%) and 73% (from 7.3% to 13.0%) among girls. Current use of any tobacco (cigarettes or waterpipe) increased 54% (from 22.1% to 34.1%) among boys and 93% (8.1% to 15.6%) among girls. Although the resurgence of waterpipe use is a fairly recent phenomenon [7] among adolescents in Jordan (as in other Middle Eastern countries; [4] its use is now more common than cigarettes and may be increasing more rapidly among girls than boys.

A large body of literature demonstrates that perceptions about health risks influence cigarette smoking among adolescents (e.g., Aryal *et al.* [14]; Mantler [15]). Studies have mainly focused on adolescents’ perceived risks or benefits of smoking [16,17], showing that individuals who perceive fewer risks and greater benefits of smoking are more susceptible to initiation [16,18]. For example, Song and colleagues [18], found that among a cohort of 395 9th graders followed for two years, those with the lowest perceptions of either short- or long-term smoking-related risks were approximately three times more likely to start smoking than those with the highest perceptions of risk. Likewise, adolescents with the highest perceptions of smoking-related benefits were more than three times as likely to initiate. Among a group of more than 700 adolescent non-smokers, health-related motives, such as fear of developing cancer and the desire to maintain physical fitness, were the most important and frequently mentioned motives for not smoking [19]. Halpern-Felsher and colleagues [16] reported that adolescent cigarette smokers, compared to non-smokers, estimated their chance of experiencing smoking-related negative health outcomes as less likely. 

Similar to findings among adolescent cigarette smokers, there is a growing body of evidence that adolescent and young adult waterpipe smokers are less likely than non-users to perceive waterpipe to be harmful to health or addictive [9], and that those who perceive greater benefits, and fewer risks from waterpipe smoking are more likely to plan to initiate [20]. While studies have examined adolescent perception of health as a consequence of tobacco use, none have examined the possibility that tobacco smoking is a result of adolescent perceptions of health. 

The primary purpose of this study was to estimate the prevalence of current tobacco smoking (cigarettes and waterpipe) among school students aged 11 to 18 years in one of the major governorates of Jordan and to examine the relationship between tobacco use and adolescents’ perceptions of health.

## 2. Methodology

The current report is a part of a larger project and dataset about tobacco use among Jordanian adolescents. The methodology has been reported previously [21]. 

### 2.1. Design, Participants, and Setting

The current study used a cross-sectional design that involved administration of a questionnaire by a trained research assistant. The study was conducted from February to June 2012 in Zarqa, one of Jordan’s central governorates, located east of the Capital Amman. Zarqa is considered the second largest industrial and the third most densely populated governorate in the country [22]. The large size and geographic heterogeneity of Zarqa allowed us to include in the sample adolescents from urban, suburban, and rural areas. The Governorate has three educational districts which have around 75 schools in total. These schools are categorized as Basic Schools that include grades 1–10 and High Schools that include grades 11 and 12. The researchers only sampled students in grades 6, 8, 10, and 12. Power analysis was conducted to determine the required sample size. Setting the significance level at 5% and power at 80%, and estimating the prevalence of tobacco smoking among males and females as 27% and 16%, respectively, the minimum sample size required to detect a difference between males and females was estimated to be a total of 680 participants.

### 2.2. Recruitment, Permission, and Data Collection

The study protocol was approved by the Institutional Review Board of The Hashemite University (HU) and the Zarqa Governorate educational districts. The sample was recruited using a multistage random sampling method. In the first stage, schools were selected. A list of schools was obtained from each of the three educational districts. From each list, schools were randomly selected utilizing a systematic simple random sampling procedure: every 5th school was included to achieve the desired 20% of schools from the list. The schools were stratified by gender (boys’ schools and girls’ schools) to ensure representativeness of the sample. In the second stage, classrooms were sampled. From each selected school, a list of classes was obtained and classrooms were randomly selected using a simple random technique. All students in each randomly selected class was asked to participate. Students were given copies of the information sheet, which included a direct contact phone number for the Principle Investigator—PI (Author), to take home to their parents. School officials indicated that they routinely send information home to parents in this way. Parents were instructed to contact the PI directly if they did not want their child to participate in the study. All participants were asked to provide their informed assent before completing study measures. Using these sampling and recruiting strategies, 1050 children within 11 schools were invited to participate. 

### 2.3. Measures

The current study used an abbreviated version of the Arabic Adolescent Tobacco Use Composite Measure (AYTUCM) [23]. This measure includes 136 items to assess known predictors of tobacco use among adolescents. This study included items of the AYTUCM that captured prevalence and patterns of tobacco use as well as perceptions of health.

Current use of cigarettes and waterpipe was defined using the standard Centers for Disease and Prevention (CDC) definition of any smoking, even one puff, during the last thirty days, based on two questions: “How many times have you smoked a cigarette, even a puff, in the past 30 days?”, and “How many times have you smoked waterpipe, even a puff, in the past 30 days?”. Based on responses to these two questions, a single current tobacco smoking variable, “*Tobacco smoking Status*”, was created with four categories. The first category included *non-smokers*; participants who reported smoking neither cigarettes nor waterpipe. Participants who smoked cigarettes only, but not waterpipe, were defined as *cigarette smokers*. Participants who smoked waterpipe only, but not cigarettes, were defined as *waterpipe smokers*. Finally, participants who used both cigarettes and waterpipe were defined as *dual smokers*.

Two items assessed health-related perceptions. The first asked participants to indicate whether they believed it is safe to smoke tobacco for only a year or two as long as they will quit after that. Answers to this question were coded as definitely not, probably not, probably yes, or definitely yes. Based on the initial data analysis and the presence of right side skewness, the answers were collapsed and recoded as “yes” for probably yes or definitely yes, or “no” for definitely not or probably not. From here forward this variable will be referred to as *Perceived health risk*. The second item asked participants to rate their health on a 6-point scale (not at all healthy, very unhealthy, somewhat unhealthy, healthy, very healthy, most healthy ever). This variable will be referred to as *Perceived health status* throughout the paper. 

The questionnaire also included demographic data (age, gender, grade), school academic performance measured by last academic year GPA. Youth were also asked about their participation in extracurricular activities, this was measured by reporting how many time during the last year the youth participated in extracurricular activities. Participating in outside school activities were measured by reporting the number of activities they were involved in the last 30 days. Youth were asked to report the number of closest friends who use tobacco (cigarettes and/or waterpipe). 

### 2.4. Data Analysis

The Statistical Package for the Social Science (SPSS) version 17 (IBM, Armonk, NY, USA) was used to analyze the data. Analysis of frequency (chi-square test) and comparison of means (One-Way ANOVA) were used to determine the relationship between health perceptions and current smoking status. Multivariable regression analyses (linear and logistic) were performed to determine the associations of perceived health risk and perceived health status on current tobacco smoking status, adjusting for potential confounders, including gender, age, grade, participation in extracurricular activities, participation in outside school activities, last year school achievement, number of siblings, and number of closest five friends who smoke cigarettes and waterpipe. For these analyses, the four-category current smoking status variable (non-smoker, cigarette-only smoker, waterpipe-only smoker, and dual smoker) was dummy-coded using non-smokers as the referent. Significance for all analyses was set at *p* < 0.05. 

## 3. Results

Of the 1050 distributed questionnaires, 993 school students were included in the final analysis. Fifty seven incomplete questionnaires were excluded. Students’ age ranged from 11 to 17 years, (mean ± SD = 14.7 ± 1.9 years). Females comprised 54% of participants (*n* = 534), and the majority of participants were Jordanians (97%). See Table 1 for sample characteristics. 

### 3.1. Tobacco Use

More than one third of students used at least one form of tobacco (either cigarettes or waterpipe; *n* = 355, 35.7%), with 2.6% (*n* = 26) smoking only cigarettes, 7.0% (*n* = 70) smoking only waterpipe, and 26.1% (*n* = 259) being dual users of both cigarettes and waterpipe. There was a significant overall difference in the distribution of responses for tobacco use by gender according to chi square analysis (Table 2), with similar percentages of boys and girls being cigarette-only or waterpipe-only users, but girls being almost twice as likely as boys to be dual users (34.3% and 17.0%, respectively). 

**Table 1:** Sample Characteristics (*N* = 993).

*Variable*	*Number*	Percent *
**Age in years**		
11–12	189	19.0
13–14	221	22.3
15–16	397	40.0
17–18	186	18.7
**Gender**		
Male	459	46.2
Female	534	53.8
**Grade**		
6th	189	19.0
8th	221	22.3
10th	397	40.0
12th	186	18.7
**Paternal Occupation**		
Blue collar	359	36.1
White collar	485	48.8
Unemployed	177	17.8
**Number of siblings**		
Equal to or less than 6 siblings	521	52.5
More than 6 siblings	441	44.4
**Extracurricular Activity involvement in last 30 days**		
I did not participate	427	43.0
One time	203	20.4
Two or more times	360	36.3
**Outside School Activity involvement in the last 30 days**		
I did not participate	415	41.8
One	170	17.1
Two or more	403	40.6
**Last year’s GPA**		
Excellent (90–100)%	239	24.1
Very good (80–89)%	281	28.3
Good (70–79)%	282	28.4
Poor 69 or Less	189	19.0
**Having one or more of their five closest friends who use waterpipe?**		
Yes	435	43.8
No	558	56.2
**Having one or more of closest five friend who use cigarettes**		
Yes	417	42.0
No	576	58.0

* total values may not add up to the exact % due to missing values.

**Table 2:** Association between participants’ gender and tobacco smoking status.

Tobacco Smoking Status	Total *N* (%)	Gender		*p*-value *
		Male *N* (%)	Female *N* (%)	
Non-smokers	638 (64.2)	332 (73.1)	298 (56.1)	<0.001
Cigarette smokers	26 (2.6)	13 (2.9)	13 (2.9)	
Waterpipe smoker	70 (7.1)	32 (7.0)	38 (7.2)	
Dual smoker	259 (26.1)	77 (17.0)	182 (34.3)	
**Total**	993(100)	454 (100)	539 (100)	

* (*X*^2^) test.

### 3.2. Relationship between Tobacco Use and Health-Related Perceptions

In a bivariate (chi-square) analysis, significant association was observed between perceived health risk and tobacco use status (*p* = 0.006) Table 3. Comparison of means (One-Way ANOVA) was used to determine the relationship between *Perceived health status* and current smoking status. The results indicated a significant difference between mean perceived health status and tobacco smoking status (*p* = 0.037) (Table 4). 

**Table 3:** Association between participants’ perceived health risk and tobacco smoking status.

Tobacco Smoking Status	Total *N* (%)	Perceived Health Risk		*p*-value *
		No *N* (%)	Yes *N* (%)	
Non-smokers	638 (100%)	389 (61.0%)	249 (39.0%)	0.006
Cigarette smokers	26 (100%)	15 (57.7%)	11 (42.3%)	
Waterpipe smoker	70 (100%)	38 (54.3%)	32 (45.7%)	
Dual smoker	259 (100%)	125 (48.3%)	134 (51.7%)	

* (*X*^2^) test.

**Table 4:** Association between participants’ perceived health status and tobacco smoking status.

Tobacco Smoking Status	Perceived Health Status		*p*-value *
	Mean	SD	
Non-smokers	3.22	±1.63	0.037
Cigarette smokers	3.00	±1.32	
Waterpipe smoker	3.25	±1.69	
Dual smoker	2.91	±1.18	

* (One way-ANOVA).

Multivariable logistic regression model was performed to examine the relationship between *Perceived health risk* and current tobacco smoking status after controlling for the effect of participants’ socio-demographics and number of closest five friends who smoke cigarettes or waterpipe (Table 5). Dual tobacco smokers were 2.5 times as likely to think that smoking for two years is not harmful to health, as long as they stop afterwards, compared to non-smokers (OR = 2.4, C.I. = 1.7–3.4). Similar patterns were found for both cigarette-only and waterpipe-only smokers (OR = 1.90 and 1.50, respectively) but these differences were not statistically significant.

**Table 5:** Adjusted effect of tobacco smoking status on perceived health risk (logistic model) **.

Variables in the Model	Adjusted^ OR	95% C.I ^!^
Age (in years)	0.90	0.82–0.97
Gender (Female)	1.21	0.90–1.63
Participating in extracurricular activities		
Do not participate	Ref	
Once per week	0.75	0.56–1.04
Twice or more per week	0.65	0.45–0.94 *
Participating in outside school activities		
I did not participate	Ref	
One	0.95	0.70–1.30
Two or more	1.03	0.70–1.52
School achievement		
Excellent	Ref	
V. good	1.10	0.71–1.70
Good	0.70	0.64–1.36
Poor	1.00	0.65–1.46
Tobacco smoking status		
Non-smoker	Ref	
Cigarette smoker	1.72	0.74–4.00
Waterpipe smoker	1.47	0.86–2.50
Dual smoker	2.51	1.74–3.63 *
Number of Siblings		
Equal to or less than 6 siblings	Ref	
More than 6 siblings	0.75	0.57–1.00 *
Number of closest five friends who smoke cigarettes	1.15	0.68–1.95
Number of closest five friends who smoke waterpipes	1.66	1.00–2.74

Notes: ^!^ C.I.: confidence interval. * *p* < 0.05; ** Perceived health risk was coded as 0 = No and 1 = Yes.

Multivariable linear regression model was performed to examine the relationship between *Perceived health status* and current tobacco smoking status after controlling for the effect of participants’ socio-demographics and number of closest five friends who smoke cigarettes or waterpipe (Table 6). No significant difference of perceived health status was found between non-smokers and cigarette-only smokers and waterpipe-only smokers. However, dual smokers perception of self-rated health status was significantly lower than non- smokers (b = −0.33, C.I. = −0.59–−0.07). 

## 4. Discussion

Concern is increasingly being expressed about the growing popularity of waterpipe use, particularly among adolescent girls [24,25]. Our results confirm studies from adolescents in other Arab countries showing that waterpipe use is now more common than cigarettes. Further, to our knowledge, this is the first study in an Arab country to document greater tobacco use among adolescent girls than boys. Our results show that among a representative, school-based sample of adolescents in Zarqa, Jordan, girls were as likely as boys to currently use only cigarettes or waterpipe, but twice as likely to be dual (cigarette plus waterpipe) smokers (34% *vs.* 17%). Dual tobacco smokers were more likely than single tobacco smokers and non-smokers to perceive that it is safe to use tobacco as long as the individual will stop after two years compared to non-smokers. Moreover, dual smokers had the lowest score on the self-reported *Perceived health status* among all tobacco users in this study. 

**Table 6:** Adjusted effect of tobacco smoking status on perceived health status (linear model) **.

Variables in the Model	Adjusted^ Effect (β)	95% C.I. ^!^
Age (in years)	0.08	0.03–0.14 *
Gender (Female)	−0.030	−0.24–0.18
participating in extracurricular activities		
Do not participate	Ref	
Once per week	0.08	−0.18–0.35
Twice or more per week	−0.12	−0.35–0.11
participating in outside school activities		
I did not participate	Ref	
One	−0.02	−0.30–0.27
Two or more	0.08	−0.16–0.30
School achievement		
Excellent	Ref	
V. good	−0.16	−0.43–0.11
Good	−0.36	−0.64–−0.09 *
Poor	−0.50	−0.81–−0.18 *
Tobacco smoking status		
Non-smoker	Ref.	
Cigarette smoker	−0.28	−0.91–0.36
Waterpipe smoker	0.05	−0.34–0.44
Dual smoker	−0.33	−0.59–−0.07 *
Number of siblings		
Equal to or less than 6 siblings	Ref	
More than 6 siblings	−0.02	−0.21–0.18
Number of closest five friends who smoke cigarettes	−0.12	−0.50–0.25
Number of closest five friends who smoke waterpipes	0.22	−0.14–0.58

^!^ C.I.: confidence interval. * *p* < 0.05; ** Perceived health status was measured on a 6-point scale of 0–5.

Several previous studies of Arab adolescents, which similarly defined current tobacco smoking as “smoking, even one puff, during the last thirty days”, have reported higher prevalence of both cigarette and waterpipe smoking for boys than girls [4,10,26,27]. For example, Christophi *et al.* [27] the prevalence of current cigarette smoking among school students was 13% among boys and 7% among girls in middle schools, and 36% among boys and 23% among girls in high schools. In a current study McKelvey and colleagues [28] followed a sample of 7th and 9th grade Jordanian school students over two years to study their tobacco smoking (cigarettes and waterpipe) trends over time. According to their results an increase of cigarettes, waterpipe, and dual tobacco smoking was found among youth. This increase was significantly higher among boys than girls for all three tobacco smoking categories. However, other studies have reported no difference of tobacco smoking especially waterpipe among boys and girls [13,29]. For example, the El-Roueiheb and colleagues [29] study shows that men are more likely to smoke cigarettes and to be dual users, but women are more likely to be waterpipe-only users. 

Reasons for the high rate of dual smoking among girls in our study are not known. While it is possible that characteristics of our sample of adolescents are different from other samples in Jordan and other Arab countries, this does not seem likely given that it was derived randomly to be representative of a large governorate. Previous studies in Jordan had only included youth who are aged 13 to 18 years such as the GYTS and Mzayek *et al.* [13], whereas our study included a wider age range (11 to 18 years). Moreover, the current study included adolescents from urban, suburban, and semirural areas. Previous studies mainly included youth from urban areas [13,28]. Another possibility is that this finding reflects a rapidly changing demographic pattern of tobacco use among male and female adolescents. Previous studies found that while cigarette smoking by women in most Arab countries was unacceptable, waterpipe smoking by females of all ages is considered a socially acceptable behavior [6]. It may be the case that the acceptability of waterpipe among girls and young women in traditional societies serves as a “foot in the door” for greater acceptability of cigarette use. 

Another potential driver of a demographic change in tobacco use patterns may be gender differences in the development of dependence. Given that adolescent tobacco users may show signs of dependence even when smoking rates are low and sporadic [30], and that girls develop signs of nicotine dependence more quickly after initiating tobacco use than boys [31], higher dual tobacco use in girls may indicate that once waterpipe use is initiated (which typically occurs earlier than initiation of cigarettes; [32] they are more likely than boys to use cigarettes as an alternate nicotine source to avoid withdrawal or gain positively reinforcing effects of nicotine that are part of a nicotine dependence syndrome. This is speculative, and further studies are needed to assess the trajectory of nicotine dependence among adolescent waterpipe users. 

These findings call for the need to implement prevention and cessation intervention programs in middle and high schools. For example, Pbert and colleagues [33] found that implementing a brief smoking-cessation counseling intervention using cognitive behavioral strategies and delivered by school nurses within the school health setting was effective in promoting short-term abstinence from cigarette smoking among adolescent smokers. Additionally, in a country where not every school has a school nurse among it’s personnel such as Jordan, these results provide strong evidence for Jordanian school officials and health decision makers to develop policies that emphasis the need for community health nurses in neighboring Health Centers to work closely with the schools to monitor the problem of tobacco smoking among adolescents and to develop and implement prevention programs. The observed gender difference in smoking patterns suggests that determinants may be gender-specific, calling for interventions that are sensitive to these differences. 

The current study indicated a significant result of the effect of tobacco smoking status on adolescent self-reported perception of health. While the majority of studies in the literature examine health risk and benefits of tobacco smoking to predict adolescent smoking status or intentions [14,15,18] our study examined the association between adolescent tobacco smoking and their perception of health. These findings could be contributed to the lack of adequate knowledge and awareness of the harmful effects of tobacco smoking provided by the curricula [6,34]. Additionally, adolescent may hold the misconception that waterpipe tobacco smoking is less harmful than smoking cigarettes [9,35]. Study findings indicated that smokers, particularly dual-smokers, were more likely to view tobacco smoking with no health risks. These findings can be contributed to youth health beliefs that tobacco smoking imposes no risk to their health. Harries and colleagues reported that current smokers were less likely to agree that smokers live shorter lives and that smokers are more likely to get heart disease. These smokers also perceived their own health risks as less severe than other smokers [36]. Another reason could be the association between perceived physical health risks and benefits of tobacco smoking and youth smoking status. Studies found a negative correlation between perceived risks of smoking, and a positive correlation between perceived benefits of smoking with youth smoking behavior [16,18]. Study results make it possible to make inferences that adolescent who smoke are more likely to have low perception of health than non-smokers. Interestingly dual smokers have the lowest perception of health of all smoking profile categories indicating that the more tobacco products used by adolescent the lower the health perception is. These findings are very important in understanding the problem of tobacco smoking among adolescent and it provide a vital clue for health professionals who works in cessation programs. Additionally, this finding can provide community and school nurses with a significant finding to target adolescent with smoking cessation programs. 

## 5. Conclusions

In conclusion, these data confirm previous reports that waterpipe use is now more prevalent than cigarette use among Arab adolescents, and show very high rates of dual use of waterpipe and cigarettes, especially for girls. Dual use was twice as high in girls as boys, a gender difference which has not been observed previously. Of concern, adolescent tobacco users, especially dual users, were more likely than non-users to underestimate the harmful effects of tobacco use, thinking that it is safe to use tobacco if the individual will stop after two years. Of course, it is well known from the cigarette smoking literature that despite intentions to only smoke for a limited amount of time, once regular use is initiated, quitting becomes very difficult. Tobacco users in this study, compared to non-smokers, perceived themselves not to be in as good health. Although poor self-reported health is common among established, adult smokers (e.g., Ward *et al.* [37]) it is worrisome that similar patterns are observed here for very young individuals who have smoked for only a short time. This finding emphasizes the lack of awareness of the harmful effects of tobacco use, and need for prevention programs to address this deficit knowledge. In addition, our findings illustrate the need for health professionals, community and school nurses to play a vital role in developing health campaigns to reach and educate adolescent, their families, teachers and school systems regarding the recognized health risks of tobacco smoking.

## 6. Study Limitations

Limitations of the study should be noted. Our sample was drawn from one governorate which may limit the generalizability of our findings across Jordan. However, it is safe to say that our sample characteristics are similar to the adolescent population in Jordan as a whole. In addition, reported use of tobacco was based solely on self-report. However, self-reported smoking in survey studies has consistently been shown to be valid in adolescents when confidentiality is assured [38]. Another limitation that future studies can overcome is measuring *Perceived Health Risk* and *Perceived Health Status* with questionnaire consisting of multiple consistent items since they represent latent variables and one item may not be sufficient to define such concepts. On the other hand, the study has several strengths, including a large random sample and a high response rate. 
